# Can Occupational Safety and Health Preventive Measures Taken by the Employer Influence Sleep Disturbances in Teleworkers? Results from the Quantitative Study on Working Life with COVID-19 in Latvia

**DOI:** 10.3390/brainsci14070684

**Published:** 2024-07-08

**Authors:** Linda Matisāne, Diāna Inga Paegle, Linda Paegle, Lāsma Akūlova, Monta Matisāne, Ivars Vanadziņš

**Affiliations:** Institute of Occupational Safety and Environmental Health, Rīga Stradiņš University, Dzirciema 16, LV-1007 Riga, Latvia; dianainga.paegle@rsu.lv (D.I.P.); linda.paegle@rsu.lv (L.P.); lasma.akulova@rsu.lv (L.A.); monta.matisane@rsu.lv (M.M.); ivars.vanadzins@rsu.lv (I.V.)

**Keywords:** telework, distance work, occupational health and safety, sleep, quality, sleep disturbances

## Abstract

This research on sleep disturbances emerged during the COVID-19 pandemic. Our study investigated the association between self-reported sleep disturbances among teleworkers and the preventive measures employers took to improve their working environment. Answers obtained via a web survey gathered from 1086 teleworkers (517 in the spring of 2021 and 569 in the spring of 2022) were analysed. The odds of self-reported sleep disturbances were significantly higher for all preventive measures in the group of respondents reporting a lack of a particular measure. The highest odds ratios were observed for the statement “My employer identified conditions where I am teleworking” (adjusted OR = 2.98, 95% CI 2.10–4.23) and “Online team-building events were organised” (adjusted OR = 2.85, 95% CI 1.88–4.35). The results of our study have revealed that workplace interventions that serve as a mediator for sleep disorders, even if they are not directly targeted at managing sleep disturbances or stress, can reduce the number of teleworkers reporting sleep disturbances. According to our knowledge, this is the first study reporting the effectiveness of employer interventions that help teleworkers manage their sleep disturbances.

## 1. Introduction

As a response to the global COVID-19 pandemic, the first step, depending on the local situation, was to reduce the spread of the SARS-CoV-2 virus [1]. A common thread among these measures was the rapid and widespread transition to telework, which also caused adverse effects and challenges for employers [2,3]. For example, compared to the general population and those workers who did not work remotely, teleworkers experienced significantly increased levels of stress and sleep problems [4,5,6]. High levels of stress and distress have also attracted the interest of researchers, especially with the focus on psychological distress; e.g., a systemic review in 2023 identified that psychological distress in teleworkers was covered in 15 out of 19 studies included in the review if compared to only 5 out of 19 studies which were devoted to physical distress [7]. 

There are many reasons behind the increased stress in teleworkers [8,9,10]. For example, there are specific cognitive and psychological stressors characteristic of telework:

(1) Teleworking is likely to have analytically demanding tasks [9];
(2) The lack of support for teleworkers [11];
(3) Cyberbullying [7];
(4) The lack of the ability to disconnect [7];
(5) The lack of appropriate ergonomic arrangements for telework [7];
(6) The perceived lack of privacy [12,13];
(7) The isolation that comes from remote work [6];
(8) Adaptation difficulties in different organisational contexts or due to the implementation of new work methodologies [6], etc.

In general, stress is strongly linked to sleep disturbances; it can affect sleep quality, leading to further mental and physical health issues, including sleep disturbances [13,14,15]. Thus, measures to alleviate stress have the potential to improve sleep disturbances [16,17]. 

Sleep disturbances (altered sleep patterns and specific sleep-related symptoms, e.g., disrupted sleep, falling asleep unintentionally, difficulties falling asleep/staying asleep, later bedtimes, more nightmares, and abnormal sleep rhythms) in the general population worsened during the COVID-19 pandemic [18,19,20]. Sleep disturbances during the pandemic garnered such attention that the term “coronasomnia” was coined, with worsening sleep quality and duration identified as the most common psychological morbidity amidst the COVID-19 pandemic [21,22]. These disturbances were reported consistently by respondents in 49 countries, highlighting the global impact of the issue [23]. 

Interestingly, when comparing teleworkers, regular workers, and unemployed individuals during the lockdown of the first wave of the COVID-19 pandemic, a protective effect of teleworking against the development of sleep disturbances was observed. The ability to maintain a regular work routine and a reduced risk of COVID-19 exposure may explain this positive impact on sleep health [24]. Teleworkers who enjoyed greater flexibility and could adapt their work schedules to sleep/wake rhythms reported longer sleep durations [24,25]. Moreover, teleworkers during the COVID-19 pandemic reallocated their former commuting time to increase their sleep time, further enhancing their sleep health [25]. 

One of the consequences that subjects with sleep disturbances exhibit compared to those who do not have sleep disturbances is a significantly higher presence of low cognitive performance (23.7% vs. 16.2%, *p* < 0.001) [26]. The link between cognitive performance and productivity is well established, contributing to an employee’s overall performance and organisational success [27]. In addition, sleep disturbances, often associated with sleep loss, can lead to fatigue and reduced job efficiency [20,28]. Furthermore, sleep loss can result in absenteeism and presenteeism, reduced work productivity, impatience with co-workers, avoidance of social interactions, and accidents at work [20,28]. 

Research on sleep disorders and telework has revealed a complex picture, with both positive and negative effects of telework on sleep disorders [2,29]. However, it is clear that due to stressors doubling the effect with harmful long-term consequences, the stress in teleworkers requires attention with a focus on the emotional and technical support of teleworkers [7,30,31]. This highlights the pressing need for further investigation and especially assessment of interventions to ensure the well-being of teleworkers [18,32]. In such a context, the role of employers is prominent in improving the work environment of teleworkers; however, employer-initiated measures have not been evaluated [4]. Our study aimed to assess the association between self-reported sleep disturbances and occupational health and safety preventive measures provided by employers that might provide evidence for tailor-made interventions at the company level that can improve sleep quality in teleworkers. Thus, our study answers the need to explore interventions that could be applied in various telework settings [33]. 

## 2. Materials and Methods

**Figure 1:**
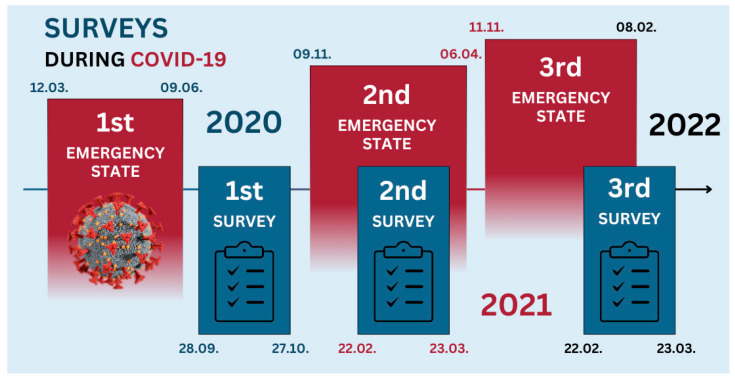
Timeline of emergency states and surveys.

A survey on working life with COVID-19 in Latvia was organised in three waves, and a cross-sectional study design was used in all waves. A survey as an online tool was used to quickly gather information from workers on measures taken by their employers to arrange healthy and safe working conditions in case of telework during the first (in force between 12 March and 9 June 2020) and the second (in force between 9 November 2020 and 6 April 2021) emergency states of the COVID-19 pandemic. The third emergency state in Latvia was declared from 11 October 2021 until 28 February 2022. The answers covering the experience of the first emergency state were gathered between 28 September and 27 October 2020. However, this questionnaire did not cover questions related to sleep disturbances; therefore, the results are not included in this article. The answers covering the second emergency state of the pandemic were gathered between 22 February and 23 March 2021 and cover both aspects—preventive measures taken by the employer and sleep disturbances reported by the workers. A year later, the third survey wave was organised between 21 February and 23 March 2022 (Figure 1).

The Ethics Commission of Rīga Stradiņš University granted ethical approval for the study. For the 1st wave, the protocol No. 6-1/08/16 from 2020 was issued, followed by protocol No. 22-2/303/2021 from 2021 for the 2nd wave and protocol No. 2-PĒK-4/120/2022 from 2022 for the 3rd wave.

### 2.1. Recruitment and Data Collection

The web survey was designed to ensure a diverse and inclusive sample. A non-probability sampling method was applied, with survey participants recruited using snowball sampling, social media advertisements, and direct emails to share the questionnaire’s web link in Latvia. The survey was open to every person with internet access, with no targeted recruitment from specific sectors. This inclusive approach ensured that the samples represented the working population from all sectors. The survey data were gathered and managed using the REDCap (Research Electronic Data Capture) tool.

At the onset of the web survey, filtering questions were meticulously applied to recruit only paid workers employed during the second and third emergency states. The exclusion criteria were carefully defined, including working without salary in family businesses, working without salary on family farms, being on maternity leave, being unemployed, being only retired, being a housewife, and being only schoolchildren or students or self-employed during the survey period. This rigorous approach ensured the precision of the survey’s target population.

While designing a survey, the survey sample size was calculated using a 5% margin error, 99% confidence intervals, a 50% response rate, 889,226 employed persons in Latvia in 2020 (for the 2nd wave of the survey) and 892,646 employed persons in Latvia in 2021 (for the 3rd wave of the survey) [34], resulting in 666 persons in both surveys. To increase the probability of finding statistically significant results and considering the planned time frame of the survey, the authors decided to make the web link available for one complete calendar month or until the moment when there were 1000 filled answers, whichever occurred first. In both cases, the web survey links were locked on the following day of the workday after 1000 respondents had answered all the survey questions.

In total, 1722 persons responded to the questions during the second wave and 2005 during the third. However, only 1027 respondents answered all questions in the survey conducted in 2021 (study adherence—59.6%) and 1240 in 2022 (study adherence—61.8%). Data on the number of clicks on the web survey landing page are unavailable. An additional three persons had to be excluded from the analysis to apply weights due to a lack of information in their answers (e.g., unwilling to specify gender or age). 

**Figure 2:**
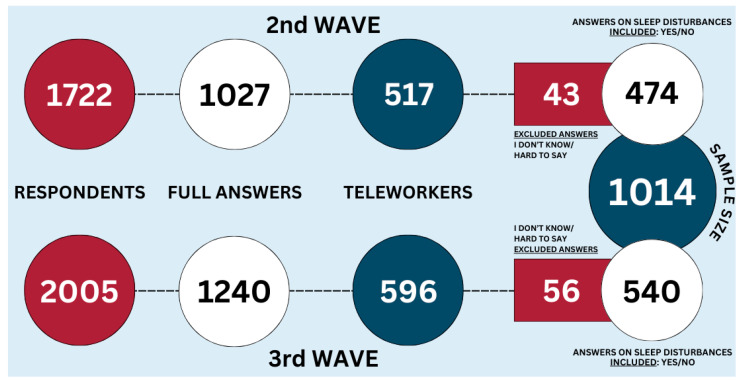
Flowchart of the sample of teleworkers used for this analysis.

For this study, we excluded several groups of respondents from further analysis. We excluded those who were not working during the 2nd and 3rd emergency states, as they could not report on the preventive measures taken by their employers. This included individuals who lost their jobs and did not find new employment during the 2nd and 3rd emergency states, and those on state-paid downtime throughout the emergency state period. Additionally, we excluded individuals who were not teleworking, as our study specifically focused on teleworkers. It resulted in a sample of 517 respondents reporting teleworking during the 2nd wave of the survey and 596 during the 3rd wave (Figure 2).

During the 2nd wave of the survey, 14.9% (*n* = 77) of the respondents had teleworking experience already before the COVID-19 pandemic. The majority, 60.0% (*n* = 310), started telework during the 1st emergency state, and 25.1% (*n* = 130) began during the 2nd emergency state. The average age of these respondents was 43.0 (SD = 10.9, min 20, max 70) years; 24.0% were males and 76.0% were females. For the 3rd wave of the survey, 16.3% (*n* = 93) of respondents had teleworking experience before the COVID-19 pandemic, and 87.3% (*n* = 476) started teleworking during the pandemic. The average age of the respondents who finished the questionnaire was 45.3 (SD = 11.3, min 22, max 74) years, and 18.5% were males and 81.5% were females. A detailed description of the study sample included in the analysis for this research with applied weights is available in Appendix A (Table A1). At the beginning of the web survey, written information on the purpose of the study was provided. Therefore, voluntarily proceeding to the questions, participants agreed to participate in the survey.

### 2.2. Instrument

A group of experienced researchers initially drafted the questionnaire of the 1st wave led by one of the authors (L.M.), which was then evaluated by two other experts (including one of the authors—I.V.) and tested by three different experts (authors—L.P., L.A.). Based on the received comments, the instrument was improved and sent for review to the Ministry of Welfare, which was the primary stakeholder to use the obtained results from the project “Life with COVID-19: Evaluation of Overcoming the Coronavirus Crisis in Latvia and Recommendations for Societal Resilience in the Future” (VPP-COVID-2020/1-0013). After this approval, the questionnaire was programmed and tested for readability, consistency of style, formatting, and clarity of the language by five independent persons who were not involved in the study and had no background related to occupational health and safety. For the 2nd and 3rd waves, a similar principle was followed. Based on the questionnaire of the 1st wave, one of the authors (L.M.) amended the questionnaire because of the changing needs and practises. Then, the instrument was evaluated, tested, improved, reviewed, programmed, and tested once again for readability, consistency of style, formatting, and clarity of the language.

The questionnaire was adapted to the respondents’ particular situations through filters and rooting. For example, the telework section was asked only to those who reported that their work could be carried out from a distance and that they worked from home during the COVID-19 pandemic.

### 2.3. Study Variables

To obtain results comparable for all three waves of the survey, a similar set of eleven statements on different preventive measures offered by employers to support workers doing their jobs from home was used. The preventive measures included in the questionnaire were selected to cover general or COVID-19-related national legal requirements (e.g., on stay-at-home policy, workplace risk assessment, occupational health and safety training) in force during the emergency states caused by COVID-19. In addition, measures provided by the employers in Latvia which had been identified as good practise examples by the State Labour Inspectorate and the Institute for Corporate Sustainability and Responsibility and published on the national occupational health and safety website www.stradavesels.lv on 8 May 2020 were included in the questionnaire [35]. All statements are given in Appendix A, Table A2 and Table A3. For each statement, several answers were possible: “It was necessary and was provided in all cases”, “It was necessary, but was provided only in some cases”, “It was necessary, but was not provided”, “It was not necessary and was not provided” (in this article, referred as “provided”, “partly provided”, “not provided”, and “not needed”), and “I don’t know/hard to say”. To calculate odds ratios and 95% confidence intervals for the association between self-reported sleep disturbances and the particular preventive measure, two groups of respondents were created: (1) teleworkers who reported that their employer provided the particular preventive measure in all cases and (2) teleworkers who reported that their employer provided the particular preventive measure only in some cases or did not provide the particular preventive measure at all, although it was needed. The distribution of all answers is given in Appendix A, Table A2 (pooled analysis) and Table A3 (by waves). Self-reported sleep disturbances were identified by the question “When working remotely, did you experience sleep disturbances related to the new working and living environment?” Several answers were possible: “Yes”, “No”, and “I don’t know/Hard to say”. Respondents who reported “I don’t know/hard to say” were considered missing values and excluded from the analysis (43 in the 2nd and 56 in the 3rd wave). No further information was asked related to sleep disturbances (e.g., sleep duration or worsening of sleep if the disturbances are specified with a diagnosis or confirmed by a physician), which is a limitation of our study (for details, see below). To calculate results with adjustment to stress, which is known to be a mediator for sleep disturbances, we used the following statement: “When working remotely, did you experience anxiety related to the new working and living environment?” It was the only question that at least somehow allowed us to assess the role of stress in mediating sleep disturbances in our study. Also, for this question, several answers were possible: “Yes”, “No”, and “I don’t know/Hard to say”.

The analysis also considered the association between self-reported sleep disturbances and several independent factors characterising the work organisation. One of these factors was the average self-reported working hours. This factor was measured by the question “How many hours per day do you work now?” Respondents were given the following options: “Less than 6 h”, “6 to 8 h”, “8 to 10 h”, “10 and more hours”, and “I don’t know/hard to say”.

Another independent factor was related to previous experience with teleworking. Respondents were asked to specify which of the mentioned statements best described their teleworking expertise: “I teleworked already before the emergency state” and “I started teleworking during the pandemic” were used to analyse the association between previous experience and self-reported sleep disturbances.

Another critical aspect for teleworkers—the need to disconnect from digital devices—was measured by the following question: “Is it important for you to be able to disconnect from digital devices outside working hours or when the assigned tasks have been fulfilled?” The respondents were able to select the answer from the following options: “Yes”, “No”, or “I don’t know/hard to say”. 

### 2.4. Statistical Analysis

The data from the 2nd and 3rd waves of surveys were pooled into one dataset for analysis. To ensure the validity of our findings, we used descriptive analyses (mean, standard deviation) and frequency analyses (percentages, distribution) to describe the data. The association between the provision of the particular preventive measure and self-reported sleep disturbances was then analysed using multinomial logistic regression and calculated as odds ratios (ORs) with 95% confidence intervals (CIs) with adjustments for gender and age as well as gender, age, education level, and work experience (aOR). Several regression models were used. For the 1st one, gender and age as confounding variables were included in the regression models; for the 2nd one, education level and work experience were added; and for the 3rd one, anxiety was added. 

Age was divided into the following groups: 18–24, 25–34, 35–44, 45–54, 55–64, and 65–74. Data weights were made by age crossed with gender (in 12 age–gender combinations) and analysed with statistical software IBM SPSS, version 27 (IBM Corporation, Armonk, New York, NY, USA). Weighting targets for the 2nd wave included population estimates for the 1st quarter of 2021; for the 3rd wave, those of the 1st quarter of 2022 were obtained by age groups and gender. Data were obtained from the Central Statistical Bureau of Latvia for both waves.

To assess and eliminate unacceptable collinearity, the variance inflation factors (VIFs) for the variables were calculated. The VIFs of independent variables in the consecutive models of the association between preventive measures, sociodemographic factors, work experience, and anxiety are reported in Table A4. Since all VIF values were below 5, collinearity among these factors was excluded.

## 3. Results

In total, 32.3% (*n* = 316) of all responding teleworkers mentioned self-reported sleep disturbances, with a variation from 37.1% in 2021 to 28.2% in 2022. Appendix A, Table A1 presents self-reported sleep disturbances in different sociodemographic groups. Table 1 provides unadjusted ORs and adjusted ORs of self-reported sleep disturbances in correlation with sociodemographic factors, with several factors characterising work organisation and anxiety, a known mediator for sleep disturbances.

Sleep disturbances were more often reported by women (60.8%) than men (39.2%). Also, the odds were higher for women (OR = 1.67). In our sample, sleep disturbances were reported slightly more often in the age group 45–54 years (30.0%, *n* = 49) compared to the nearly identical distribution in age groups above and below this range: 35–44 years (21.1%, *n* = 35), 25–34 years (20.5%, *n* = 34), and 55–64 years (21%, *n* = 35). The youngest and oldest teleworkers reported markedly lower experience of sleep disturbances: 18–24 years (5.4%, *n* = 9) and 65–74 years (2.0%, *n* = 3). No statistically significant difference in the odds of self-reported sleep disturbances was found in respondents by age group, meaning that age did not seem to affect the occurrence of sleep disturbances. Interestingly, the age group 45–54 years reported slightly more sleep disturbances (30.0%) compared to the age groups 35–44 years (21.1%), 25–34 years (20.5%), and 55–64 years (21%). The youngest and oldest teleworkers, aged 18–24 years (5.4%) and 65–74 years (2.0%), respectively, reported significantly lower sleep disturbance rates.

Work experience of 5 to 10 years (OR = 1.81), followed by 2 to 5 years (OR = 1.74), significantly increased the odds of self-reported sleep disturbances, and so did vocational secondary education (OR = 3.00). However, these categories became insignificant when adjusted for age and gender. The odds of self-reported sleep disturbances significantly increased for employees who started teleworking during the COVID-19 pandemic (OR = 2.39) and remained almost equally high after adjusting for age and gender (aOR = 2.37). Likewise, the unadjusted odds significantly increased for teleworkers who, on average, worked more than 10 h daily (OR = 2.44). These odds remained significant after adjustment (aOR = 2.61). Furthermore, the odds of self-reported sleep disturbances increased for teleworkers who identified the importance of disconnecting from digital devices (OR = 1.76, aOR = 1.67). Of those respondents who identified a need to disconnect from digital devices, 63.1% (*n* = 147) were women. 

When looking at the association between the reported anxiety and sleep disturbances, we found an extraordinarily high OR that highlights the role of stress in mediating sleep disturbances (OR = 21.05, aOR = 21.50). 

The odds of self-reported sleep disturbances were increased across all preventive measures, and most of the findings are highly significant (*p* < 0.001). They remained significant after adjustment for age and gender and after adjustment for age, gender, education, and work experience with the exclusion of a provision for a computer and other IT equipment, which became insignificant when adjusted for. Because of our findings on the strong association between anxiety and sleep disturbances, we also decided to adjust the OR for anxiety. In this case, the odds for sleep disturbances remained increased and significant only for information on how to arrange an ergonomic workstation, the identification of teleworking conditions, and training on stress management (for details, see Table 2 and Appendix A: Table A2 and Table A3).

## 4. Discussion

Our research findings demonstrate a significant association between all preventive interventions carried out by employers at the workplace level and self-reported sleep disturbances. This finding is consistent with the research of other authors [7,36]. However, three of the preventive measures (information on how to arrange an ergonomic workstation, identification of teleworking conditions, and training on stress management) seem to have independent effects on the OR of sleep disturbances. Further, we tried to explain why and how our analysed preventive measures can influence stress and, thus, sleep disturbances.

The earlier research findings suggest that lack of the required IT equipment in teleworking and problems with home-based computers are identified as typical stress factors in teleworkers [37,38]. These technical disturbances are particularly distressing because they cause interruptions of telework and are not as transparent for teleworkers’ managers and colleagues as they would have been in the company offices. Therefore, provision of company-owned equipment (addressed in our first statement, “I was provided with a computer and other IT equipment”) and measures addressing how to adjust computers for teleworking best (assessed in our second statement, “I received IT support on how to adjust my computer for telework”) can reduce this stress component [37,39]. 

When teleworking, macroergonomics (how the work systems function through teleworkers together using information and communication technology (ICT) within the organisational system) plays an essential role and is determined to be crucial in supporting telework and eliminating the stress associated with a malfunction of ICT [33,40,41]. Employers’ level of addressing this problem was evaluated through our third statement: “I was trained on how to use tools and software I did not use earlier (e.g., Zoom, Microsoft Teams, etc.)”. 

The use of information and communication technologies is the basis of telework. It is associated with a particular type of stress called “technostrain”, which originates from stress due to use of technology [42,43,44]. Thus, information and communication technology facilitators, such as the information on which tools are best to use, where to receive additional support if needed, and how to tailor the use of technology to the specific occupation performed via teleworking, reduce this stress [45]. The evaluation of the actions taken by the employers within our research was based on our fourth statement: “I received support on how to do my job via teleworking (e.g., for my occupation, which tools to use, where to receive support, etc.)”. 

Previous research has also highlighted that the lack of appropriate ergonomic arrangements for telework can increase stress in teleworkers, as in the home office, teleworkers typically set up their workstations without assistance and training, sometimes even on coffee tables, ironing boards, kitchen tables, or old desks, and this can result in back pain or other problems related to physical health [23,46]. These aggravated health conditions because of poor ergonomics in home offices are also one of the stressors for teleworkers [6,7]. With our statements, we tried to assess both the availability and provision of equipment needed to arrange the workplace ergonomically (the fifth statement, “My employer provided an office table and office chair”) and the knowledge on how to arrange the workplace ergonomically (the sixth statement, “I received information on how to arrange an ergonomic workstation”). 

Our earlier findings of the first wave of the COVID-19 survey show that teleworkers who reported no workplace risk assessment (analysed by using the seventh statement, “My employer identified conditions where I am teleworking”) had increased odds of anxiety (OR = 2.83, 95% CI 1.50–5.35) [47]. When an employer identifies conditions where an employee is teleworking, this should lead to organisational support at the line manager level and reduce associated stress and sleep disturbances. It is important to stress that the statement “My employer identified conditions where I am teleworking” was used to identify if the employer has carried out a workplace risk assessment for the workplace in the home office. This is a requirement set by the Council Directive 89/391/EEC of 12 June 1989 on the introduction of measures to encourage improvements in the safety and health of workers at work (the so-called Framework Directive) stating that “the employer shall have an assessment of the risks to safety and health at work, including those facing groups of workers exposed to particular risks and decide on the protective measures to be taken and, if necessary, the protective equipment to be used” [48], which has been in force since 31 December 1992. These general provisions have been applicable in Latvia since 2002 [49]. 

Teleworking poses challenges for both employers and teleworkers. One of the challenges for teleworkers could be the increased costs of working from home due to higher internet or electricity costs, causing stress [9,50]. By using the eighth statement, “My employer compensated costs arising from telework (e.g., internet, electricity)”, we wanted to evaluate whether measures directed at reducing these concerns for teleworkers would alleviate stress and, thus, sleep disturbances.

One of the key stressors of telework is a considerable decrease in informal information sharing and informal communication, which teleworkers intensely miss [37]. Working from home can even be isolating, so it is essential to connect with the rest of the team [51]. Online team-building activities have shown to be important not only for the well-being and reduction in the stress of the employees but also for improving organisational productivity and for building the team‘s capability to adapt to the changing conditions during the COVID-19 pandemic [39,52]. The aspect of having team-building events was assessed with our ninth statement—“Online team-building events were organized (breakfast, lunch, games, etc.)”.

Research demonstrates that nearly a third of teleworkers feel stressed when dealing with their managers [53]. This underscores the crucial role managers play in the well-being of their teams. Problematic employer–employee relationships or insufficient organisational support generate stress [38,53]. With the 10th statement, “My direct supervisor was trained in online management skills”, we wanted to evaluate whether working with a manager trained in online management skills would reduce sleep disturbances due to lessened stress from a manager skilled in online communication and work management.

Research has already shown the effectiveness of workplace stress management training in decreasing stress [54]. Therefore, the inclusion of the statement “Training on stress management was provided to me” (our 11th statement) was a logical addition to the list of assessed preventive measures. 

Our research underscores the crucial role of autonomy in telework, but it also highlights a potential pitfall. The teleworkers reporting interventions that supported their autonomy, such as providing information on how to do the job better, reported significantly fewer sleep disturbances. Autonomy in telework, which includes the choice of location and scheduling, is a critical factor in enhancing productivity and reducing sleep disturbances [19,55]. However, it is essential to note that the effectiveness of autonomy can be compromised if there is a lack of clarity on how to do telework. It is where clear guidelines and support come in. They are beneficial and necessary to maintain the positive effects of autonomy and ensure the well-being of teleworkers. Interventions addressing information undersupply and isolation provide more clarity. Therefore, this explains why interventions addressing the management of remote tools, tailoring teleworking to the particular job at hand, and receiving additional support if needed appeared highly effective in reducing sleep disturbances. 

Our results demonstrate that teleworkers benefit from employer-led guidance and social support activities that strengthen their sense of control over their work (ability to execute their tasks competently) through reduced stress and enhanced motivation and growth [56]. The effect size of the response to these activities in our results speaks to teleworkers’ high need for targeted interventions. Such a need has been identified as a priority for teleworkers worldwide [2].

When looking at the results of our study in terms of limitations, we have identified several of them. One is related to using a web survey to gather respondents’ answers. Some groups of workers may be excluded from the sample by default (e.g., the elderly, people living in remote areas, and people with low education and digital literacy) [57]. However, this limitation did not influence the results much, as these people probably did not telework. In addition, our questionnaire was available only in the Latvian language, which might have caused a lower response rate from the side of the Russian-speaking population. Another study limitation is that we used a non-probability sampling method to gather survey data. The advantage of this method is the possibility to gather information fast from respondents, which was necessary because of the implementation requirements of the project “Life with COVID-19: Evaluation of Overcoming the Coronavirus Crisis in Latvia and Recommendations for Societal Resilience in the Future” [57]. To overcome this limitation at least partly and to obtain data representative of the demographic profile of the working population in Latvia, the sample was weighted based on gender and age. We could not weigh education or work experience data, as population estimates from the Central Statistical Bureau of Latvia for the study period were unavailable. 

To avoid a self-selection bias of participants interested in sleep, this study advertisement did not mention words such as “sleep”, “sleep disorders”, “sleep disturbances”, or any other similar words and their combinations. More than 50 questions were asked to the study participants, and only one was about sleep disturbances. Therefore, before answering, the participants were unaware that the questionnaire would cover this topic.

Our study did not use any specific sleep questionnaire, a typical limitation of studies investigating the psychological effects of confinement during viral outbreaks [58]. Another limitation could be related to confounders that can affect sleep disturbances. We did not consider presenteeism, absenteeism, or physical, mental, and social health history. Medical, psychological, and social disturbances affect sleep quality bidirectionally [59]. We did not evaluate categories of sleep disorders—for example, circadian-rhythm sleep–wake disorders [60]. We did not account for chronotypes in our study. Evening chronotypes, for example, do not show their characteristic vulnerability to sleep problems when teleworking [61]. However, in demonstrating an apparent effect of interventions on sleep disturbances of any genesis, our data are robust in providing future research directions and starting organisational interventions to improve sleep quality and teleworkers’ well-being, productivity, and retention. 

In addition, our study did not account for the effect of a decrease in physical activity, which increases the risk of sleep disturbances. However, the link between the two must be clarified [62]. We did not explore the extent of workspaces’ physical and cognitive separation from nonwork spaces [63]. We did not evaluate exposure to information technology. The higher the exposure to information technology, the lower the quantity and quality of sleep [64]. There could have been confounders of positive mental and physical health. Perhaps health and fitness-aware teleworkers are also more aware and receptive to health and safety guidance and instructions.

Despite these limitations, the survey provides descriptive information and valuable insights into the possible influence of employers’ preventive measures on teleworkers’ self-reported sleep disturbances during the COVID-19 pandemic. The survey findings demonstrate that employers’ actions to strengthen teleworkers’ unmet needs for support can significantly improve sleep disturbances. To our knowledge, no studies explore tailor-made interventions by employers to address the relational component of teleworking and sleep disturbances, thus impacting cognitive fitness in teleworkers. In addition, telework organisation is a topic to be addressed at the company level because the more time an individual spends working, the less time they spend sleeping, even on non-workdays [64].

## 5. Conclusions

The results of our study have revealed that workplace interventions, even if they are not directly targeted at the management of sleep disturbances, are effective in reducing sleep disturbances if these interventions address an unmet need for relatedness and management of stress in teleworkers (for instance, support on how to do a job via teleworking, training on stress management, how to arrange an ergonomic workstation, and online team-building events). Moreover, such interventions seem more effective than technical measures to provide ergonomic workplaces or compensate for costs arising from telework. In addition, workplace risk assessment, which is a legal requirement set by the EU legal framework, is a tool that should be used to tackle sleep disturbances of teleworkers. According to our knowledge, this is the first study reporting the effectiveness of employer interventions that help teleworkers manage sleep disturbances. Therefore, clinicians who support their patients who are teleworkers may suggest that they ask their employers for support and interventions.

## Figures and Tables

**Table 1:** The odds of self-reported sleep disturbances in correlation with sociodemographic factors, with several factors characterising work organisation and anxiety.

	Self-Reported Sleep Disturbances, OR (CI 95%), Unadjusted	Self-Reported Sleep Disturbances, aOR (CI 95%), Adjusted for Gender and Age
Gender		
Female	1.67 * (1.28–2.20)	-
Male	1	-
Age		
65–74 years	0.97 (0.41–2.34)	-
55–64 years	1.09 (0.56–2.13)	-
45–54 years	1.49 (0.77–2.86)	-
35–44 years	1.35 (0.71–2.59)	-
25–34 years	1.35 (0.70–2.60)	-
18–24 years	1	-
Education		
Higher education	1.61 (0.77–3.36)	1.48 (0.70–3.10)
Vocational secondary education	3.00 * (1.11–8.11)	2.68 (0.98–7.31)
Secondary school education	1	1
Elementary school education	- (excluded due to lack of respondents)	- (excluded due to lack of respondents)
Work experience		
10 years and more	1.53 (0.93–2.52)	1.59 (0.92–2.74)
5 to 10 years	1.81 * (1.06–3.10)	1.69 (0.97–2.93)
2 to 5 years	1.74 * (1.02–2.98)	1.69 0.98–2.91)
1 to 2 years	1.23 (0.61–2.44)	1.19 (0.60–2.39)
Less than 1 year	1	1
Previous teleworking experience		
Started to telework during the COVID-19 pandemic	2.39 *** (1.62–3.54)	2.37 *** (1.59–3.54)
Started to telework before the COVID-19 pandemic	1	1
Average working hours		
More than 10 h	2.44 ** (1.29–4.64)	2.61 ** (1.37–4.99)
8–10 h	1.53 (0.86–2.74)	1.64 (0.91–2.95)
6–8 h	0.81 (0.45–1.48)	0.84 (0.46–1.54)
Less than 6	1	1
Importance of the possibility of disconnecting from digital devices		
Yes	1.76 *** (1.25–2.47)	1.67 ** (1.18–2.35)
No	1	1
Reported anxiety		
Yes	21.05 * (14.59–30.37)	21.50 * (14.75–31.35)
No	1	1

The reference category for the group of respondents reporting sleep disturbances is the group of respondents who did not report sleep disturbances; * *p* < 0.001, ** *p* < 0.01, *** *p* < 0.05.

**Table 2:** The odds of self-reported sleep disturbances with occupational health and safety preventive measures provided by the employers.

Statements on Occupational Health and Safety Preventive Measures Provided by the Employers	Answers	Self-Reported Sleep Disturbances, OR (CI 95%), Unadjusted	Self-Reported Sleep Disturbances, aOR (CI 95%), Adjusted for Gender and Age	Self-Reported Sleep Disturbances, aOR (CI 95%), Adjusted for Gender, Age, Education and Work Experience	Self-Reported Sleep Disturbances, aOR (CI 95%), Adjusted for Gender, Age, Education, Work Experience and Anxiety
I was provided with a computer and other IT equipment	No	1.41 * (1.02–1.96)	1.31 (0.94–1.83)	1.34 (0.95–1.87)	1.11 (0.72–1.71)
	Yes	1	1	1	1
I received IT support on how to adjust my computer for telework	No	1.71 ** (1.17–2.49)	1.62 * (1.11–2.37)	1.66 ** (1.13–2.43)	1.48 (0.89–2.47)
	Yes	1	1	1	1
I was trained on how to use tools and software I did not use earlier (e.g., Zoom, MicrosoftTeams, etc.)	No	1.57 ** (1.14–2.15)	1.52 ** (1.11–2.10)	1.59 ** (1.15–2.20)	1.05 (0.69–1.60)
	Yes	1	1	1	1
I received support on how to do my job via teleworking (e.g., for my occupation, which tools to use, where to receive support, etc.)	No	2.11 *** (1.50–2.97)	2.05 *** (1.45–2.90)	2.14 * (1.51–3.04)	1.28 (0.81–2.02)
	Yes	1	1	1	1
My employer provided an office table and office chair	No	1.92 *** (1.29–2.88)	1.82 ** (1.21–2.73)	2.03 * (1.33–3.10)	1.34 (0.80–2.25)
	Yes	1	1	1	1
I received information on how to arrange an ergonomic workstation	No	2.59 *** (1.85–3.65)	2.54 *** (1.80–3.57)	2.68 * (1.90–3.79)	2.00 ** (1.28–3.12)
	Yes	1	1	1	1
My employer identified conditions where I am teleworking	No	3.04 *** (2.15–4.31)	2.98 *** (2.10–4.23)	2.91 * (2.05–4.15)	2.04 ** (1.31–3.18)
	Yes	1	1	1	1
My employer compensated costs arising from telework (e.g., internet, electricity)	No	2.17 ** (1.26–3.73)	2.09 ** (1.21–3.62)	2.07 *** (1.19–3.58)	0.96 (0.48–1.93)
	Yes	1	1	1	1
Online team-building events were organised (breakfast, lunch, games, etc.)	No	2.62 *** (1.74–3.93)	2.85 *** (1.88–4.35)	2.59 * (1.69–3.97)	1.21 (0.71–2.09)
	Yes	1	1	1	1
My direct supervisor was trained in online management skills	No	2.31 *** (1.48–3.62)	2.29 *** (1.45–3.62)	2.23 * (1.40–3.55)	1.17 (0.64–2.16)
	Yes	1	1	1	1
Training on stress management was provided to me	No	2.19 *** (1.56–3.08)	2.17 *** (1.54–3.07)	2.36 * (1.66–3.35)	1.64 * (1.05–2.56)
	Yes	1	1	1	1

The reference category for the group of respondents reporting a lack of preventive measures provided is the group of respondents who did receive preventive measures provided by the employers. The reference category for the respondents reporting sleep disturbances is those who did not report sleep disturbances. * *p* < 0.001, ** *p* < 0.01, *** *p* < 0.05.

## Data Availability

The original data presented in the study are openly available in the Supplementary Materials.

## Supplementary Materials

The supporting information–Excel file with labelled data can be downloaded at https://www.mdpi.com/article/10.3390/brainsci14070684/s1.

## Appendix A

**Table A1:** Sociodemographic characteristics of the web survey study sample, *n* (%).

	Distribution of the Study Sample, *n* (%)					
	Second Wave			Third Wave		
	Total Sample	Sample of Teleworkers Included in the Analysis	Reported Sleep Disturbances	Total Sample	Sample of Teleworkers Included in the Analysis	Reported Sleep Disturbances
Gender						
Female	510 (49.8%)	254 (57.1%)	102 (62.0%)	622 (50.5%)	257 (48.0%)	90 (59.3%)
Male	514 (50.2%)	191 (42.9%)	63 (38.0%)	609 (49.5%)	278 (52.0%)	61 (40.7%)
Age						
65–74 years	49 (4.8%)	19 (4.2%)	3 (2.0%)	63 (5.2%)	29 (5.4%)	9 (6.1%)
55–64 years	211 (20.6%)	86 (19.3%)	35 (21.0%)	255 (20.7%)	101 (18.9%)	19 (12.6%)
45–54 years	245 (23.9%)	111 (24.9%)	49 (30.0%)	288 (23.4%)	105 (19.5%)	27 (17.9%)
35–44 years	241 (23.6%)	105 (23.5%)	35 (21.1%)	296 (24.1%)	155 (29.0%)	52 (34.4%)
25–34 years	226 (22.0%)	105 (23.5%)	34 (20.5%)	257 (20.8%)	111 (20.7%)	38 (25.0%)
18–24 years	52 (5.1%)	20 (4.6%)	9 (5.4%)	72 (5.8%)	35 (6.5%)	6 (4.0%)
Education						
Elementary school education	2 (0.2%)	-	-	7 (0.5%)	7 (1.3%)	4 (2.5%)
Secondary school education	78 (7.7%)	27 (6.0%)	7 (4.2%)	50 (4.0%)	15 (2.8%)	3 (1.9%)
Vocational secondary education	75 (7.3%)	17 (3.8%)	7 (4.2%)	85 (6.9%)	16 (3.1%)	8 (5.6%)
Higher education	863 (84.8%)	400 (90.2%)	151 (91.6%)	1087 (88.6%)	496 (92.8%)	135 (90.0%)
Work experience						
Less than 1 year	87 (8.5%)	37 (8.4%)	9 (5.3%)	144 (11.7%)	67 (12.6%)	16 (10.6%)
1 to 2 years	93 (9.0%)	38 (8.6%)	13 (7.8%)	63 (5.1%)	32 (6.0%)	7 (4.3%)
2 to 5 years	208 (20.3%)	96 (21.5%)	34 (20.4%)	205 (16.7%)	100 (18.8%)	35 (23.2%)
5 to 10 years	205 (20.1%)	87 (19.6%)	36 (21.8%)	245 (19.9%)	109 (20.3%)	34 (22.8%)
10 years and more	428 (42.1%)	186 (41.9%)	74 (44.7%)	573 (46.6%)	226 (42.3%)	59 (39.1%)

**Table A2:** The distribution of respondents reporting sleep disturbances in relation to the implementation of preventive measures to provide a safe and healthy workplace for teleworkers, pooled, *n* (%).

Statements Describing Preventive Measures Provided by the Employer	Sleep Disturbances	Provided	Partly Provided	Not Provided	Not Needed	I Don’t Know/Hard to Say
I was provided with a computer and other IT equipment	Yes	204 (64.8%)	35 (11.1%)	41 (13.0%)	33 (10.5%)	2 (0.6%)
	No	484 (72.8%)	66 (9.9%)	63 (9.5%)	42 (6.3%)	10 (1.5%)
I received IT support on how to adjust my computer for telework	Yes	205 (65.1%)	42 (13.3%)	15 (4.8%)	48 (15.2%)	5 (1.6%)
	No	484 (72.9%)	63 (9.5%)	17 (2.6%)	93 (14.0%)	7 (1.0%)
I was trained on how to use tools and software I did not use earlier (e.g., Zoom, Microsoft Teams, etc.)	Yes	153 (48.4%)	71 (22.5%)	27 (8.5%)	60 (19.0%)	5 (1.6%)
	No	352 (53.0%)	95 (14.3%)	49 (7.4%)	153 (23.0%)	15 (2.3%)
I received support on how to do my job via teleworking (e.g., for my occupation, which tools to use, where to receive support, etc.)	Yes	116 (36.8%)	67 (21.2%)	33 (10.4%)	92 (29.1%)	8 (2.5%)
	No	286 (43.2%)	83 (12.5%)	34 (5.1%)	240 (36.2%)	20 (3.0%)
My employer provided an office table and office chair	Yes	47 (14.9%)	28 (8.9%)	120 (38.0%)	108 (34.1%)	13 (4.1%)
	No	116 (17.5%)	46 (6.9%)	144 (21.7%)	338 (51.0%)	19 (2.9%)
I received information on how to arrange an ergonomic workstation	Yes	100 (31.6%)	49 (15.5%)	71 (22.5%)	80 (25.3%)	16 (5.1%)
	No	270 (40.7%)	43 (6.5%)	81 (12.2%)	248 (37.4%)	21 (3.2%)
My employer identified conditions where I am teleworking	Yes	66 (20.9%)	39 (12.3%)	110 (34.9%)	75 (23.7%)	26 (8.2%)
	No	241 (36.3%)	50 (7.5%)	127 (19.2%)	206 (31.1%)	39 (5.9%)
My employer compensated costs arising from telework (e.g., internet, electricity)	Yes	19 (6.0%)	16 (5.1%)	204 (64.6%)	62 (19.6%)	15 (4.7%)
	No	57 (8.6%)	22 (3.3%)	278 (41.9%)	255 (38.4%)	52 (7.8%)
Online team-building events were organized (breakfast, lunch, games, etc.)	Yes	40 (12.6%)	54 (17.0%)	108 (34.1%)	82 (25.9%)	33 (10.4%)
	No	140 (21.1%)	81 (12.2%)	134 (20.2%)	250 (37.6%)	59 (8.9%)
My direct supervisor was trained in online management skills	Yes	39 (12.3%)	23 (7.3%)	72 (22.8%)	34 (10.8%)	148 (46.8%)
	No	119 (17.9%)	37 (5.6%)	86 (13.0%)	119 (17.9%)	302 (45.6%)
Training on stress management was provided to me	Yes	72 (22.7%)	47 (14.8%)	133 (42.0%)	48 (15.1%)	17 (5.4%)
	No	176 (26.5%)	76 (11.4%)	125 (18.8%)	245 (36.8%)	43 (6.5%)

**Table A3:** The distribution of respondents reporting sleep disorders in relation to the implementation level of preventive measures to provide a safe and healthy workplace for teleworkers, by year, *n* (%).

Statements Describing Preventive Measures Provided by the Employer	Year of Survey	Provided	Partly Provided	Not Provided	Not Needed	I Don’t Know/Hard to Say
I was provided with a computer and other IT equipment	2021	101 (61.4%)	16 (9.5%)	24 (14.6%)	23 (14.1%)	1 (0.4%)
	2022	103 (68.3%)	19 (12.8%)	17 (11.5%)	9 (6.2%)	2 (1.2%)
I received IT support on how to adjust my computer for telework	2021	99 (60.3%)	24 (14.4%)	8 (4.9%)	32 (19.4%)	2 (1.0%)
	2022	106 (70.2%)	19 (12.3%)	7 (4.7%)	16 (10.3%)	4 (2.5%)
I was trained on how to use tools and software I did not use earlier (e.g., Zoom, Microsoft Teams, etc.)	2021	74 (44.8%)	36 (21.6%)	15 (8.9%)	40 (24.0%)	1 (0.7%)
	2022	79 (52.4%)	35 (23.3%)	13 (8.4%)	20 (13.3%)	4 (2.6%)
I received support on how to do my job via teleworking (e.g., for my occupation, which tools to use, where to receive support, etc.)	2021	51 (31.0%)	33 (20.1%)	18 (10.7%)	59 (35.8%)	4 (2.5%)
	2022	65 (42.9%)	34 (22.2%)	16 (10.3%)	33 (21.8%)	4 (2.8%)
My employer provided an office table and office chair	2021	20 (12.1%)	11 (6.7%)	59 (35.9%)	65 (39.5%)	9 (5.8%)
	2022	27 (17.7%)	17 (11.0%)	61 (40.2%)	43 (28.6%)	4 (2.5%)
I received information on how to arrange an ergonomic workstation	2021	44 (26.5%)	22 (13.4%)	41 (24.9%)	48 (29.2%)	10 (6.0%)
	2022	56 (37.4%)	27 (17.6%)	30 (19.9%)	32 (21.1%)	6 (4.0%)
My employer identified conditions where I am teleworking	2021	36 (21.6%)	18 (10.8%)	54 (32.9%)	45 (27.3%)	12 (7.4%)
	2022	31 (20.4%)	21 (13.8%)	56 (36.7%)	30 (20.0%)	14 (9.1%)
My employer compensated costs arising from telework (e.g., internet, electricity)	2021	9 (5.3%)	11 (6.4%)	102 (61.6%)	33 (20.2%)	11 (6.5%)
	2022	11 (7.0%)	6 (3.6%)	102 (67.6%)	29 (19.0%)	4 (2.8%)
Online team-building events were organized (breakfast, lunch, games, etc.)	2021	22 (13.4%)	24 (14.6%)	59 (35.5%)	38 (22.8%)	23 (13.7%)
	2022	18 (11.9%)	30 (19.5%)	49 (32.6%)	44 (29.2%)	10 (6.8%)
My direct supervisor was trained in online management skills	2021	22 (13.1%)	12 (7.4%)	42 (25.8%)	15 (9.2%)	74 (44.5%)
	2022	18 (11.7%)	10 (6.9%)	29 (19.4%)	19 (12.6%)	75 (49.4%)
Training on stress management was provided to me	2021	28 (16.9%)	18 (11.0%)	81 (49.5%)	29 (17.7%)	8 (4.9%)
	2022	44 (29.2%)	29 (18.9%)	51 (33.8%)	18 (12.2%)	9 (6.0%)

**Table A4:** The variance inflation factors (VIFs) of independent variables in the consecutive models of the association between preventive measures, sociodemographic factors, work experience, and anxiety.

Statements on Occupational Health and Safety Preventive Measures Provided by the Employers		Gender	Age	Education	Work Experience	Anxiety
I was provided with a computer and other IT equipment	1.041	1.066	1.364	1.015	1.356	1.053
I received IT support on how to adjust my computer for telework	1.022	1.056	1.372	1.015	1.371	1.059
I was trained on how to use tools and software I did not use earlier (e.g., Zoom, Microsoft Teams, etc.)	1.044	1.043	1.397	1.028	1.388	1.079
I received support on how to do my job via teleworking (e.g., for my occupation, which tools to use, where to receive support, etc.)	1.082	1.043	1.517	1.019	1.504	1.131
My employer provided an office table and office chair	1.087	1.083	1.508	1.044	1.504	1.125
I received information on how to arrange an ergonomic workstation	1.076	1.061	1.408	1.046	1.404	1.123
My employer identified conditions where I am teleworking	1.073	1.050	1.414	1.025	1.419	1.112
My employer compensated costs arising from telework (e.g., internet, electricity)	1.053	1.041	1.274	1.035	1.261	1.099
Online team-building events were organised (breakfast, lunch, games, etc.)	1.155	1.109	1.424	1.046	1.436	1.153
My direct supervisor was trained in online management skills	1.129	1.096	1.652	1.032	1.648	1.181
Training on stress management was provided to me	1.085	1.074	1.418	1.023	1.414	1.141
